# Prevalence of renal insufficiency in elderly cancer patients in a tertiary cancer center

**DOI:** 10.1590/S1679-45082014AO3003

**Published:** 2014

**Authors:** Lucíola de Barros Pontes, Yuri Philippe Pimentel Vieira Antunes, Diogo Diniz Gomes Bugano, Theodora Karnakis, Auro del Giglio, Rafael Aliosha Kaliks

**Affiliations:** 1 Hospital Israelita Albert Einstein, São Paulo, SP, Brazil.; 2 MD Anderson Cancer Center, Houston, Texas, USA.; 3 Faculdade de Medicina do ABC, Santo André, SP, Brazil.

**Keywords:** Renal insufficiency, Aged, Neoplasms, Prevalence, Glomerular filtration rate

## Abstract

**Objective:**

To estimate the prevalence of abnormal glomerular filtration rate in elderly patients with solid tumors.

**Methods:**

A retrospective study with patients aged >65 years diagnosed with solid tumors between January 2007 and December 2011 in a cancer center. The following data were collected: sex, age, serum creatinine at the time of diagnosis and type of tumor. Renal function was calculated using abbreviated Modification of Diet in Renal Disease (MDRD) formulae and then staged in accordance with the clinical practice guidelines published by the Working Group of the National Kidney Foundation.

**Results:**

A total of 666 patients were included and 60% were male. The median age was 74.2 years (range: 65 to 99 years). The most prevalent diagnosis in the study population were colorectal (24%), prostate (20%), breast (16%) and lung cancer (16%). The prevalence of elevated serum creatinine (>1.0mg/dL) was 30%. However, when patients were assessed using abbreviated MDRD formulae, 66% had abnormal renal function, stratified as follows: 45% with stage 2, 18% with stage 3, 3% with stage 4 and 0.3% with stage 5.

**Conclusion:**

To the best of our knowledge, this was the first study to estimate the frequency of renal insufficiency in elderly cancer patients in Brazil. The prevalence of abnormal renal function among our cohort was high. As suspected, the absolute creatinine level does underestimate renal function impairment and should not be used as predictor of chemotherapy metabolism, excretion and consequent toxicity.

## INTRODUCTION

Increased life expectancy has led to a significant expansion of the elderly population. According to the Brazilian Institute of Geography and Statistics (IBGE, Instituto Brasileiro de Geografia e Estatística),^(1)^ Brazil has 13 million people older than 65 years, with an expected increase of this group to 39 million, in 2040. Since more than 60% of all cancers and 70% of all cancer-related deaths occur in patients aged over 65 years,^(2)^ the ageing population will pose a significant challenge related to oncological treatment.

When choosing the appropriate treatment for elderly patients, special attention should be drawn to some physiological changes, such as declining renal function that predisposes the elderly to greater drug toxicity.^(3)^ The frequency of renal insufficiency (RI) in cancer patients is unclear, and the studies showed a prevalence ranging from 33% to as high as 60%.^(4,5)^

It is well recognized that approximately at 70 years, renal function may have declined by 40%^(6)^ from its baseline. Thus, before initiating potentially toxic drug therapy, such as chemotherapy, renal function should be routinely evaluated. Serum creatinine (SCR) levels are commonly used in clinical practice, but that they do not give an accurate indication of renal function, particularly in the elderly, in whom it underestimates RI.^(7)^ More accurate and modern tools, such as the abbreviated Modification of Diet in Renal Disease (MDRD),^(8)^ may better estimate renal function in elderly patients, as shown in some studies that provided a reliable prediction for glomerular filtration rate (GFR) in this population.^(9,10)^

Considering that a number of chemotherapeutical agents are cleared through the kidneys and can affect renal function, and since variability in reported renal dysfunction is significant, we aimed to study the prevalence of abnormal GFR among elderly patients diagnosed with solid tumors.

## OBJECTIVE

To estimate the prevalence of abnormal glomerular filtration rate in elderly patients with solid tumors in a cancer center.

## METHODS

Retrospective study that included patients aged >65 years diagnosed with solid tumors between January 2007 and December 2011 at *Hospital Israelita Albert Einstein* (HIAE), São Paulo (SP), Brazil.

We collected the following data from the electronic organizational database: patient’s sex, age, weight, type of tumor and SCR at the time of diagnosis. The upper limit of normalcy for SCR at HIAE central laboratory is 1mg/dL. Renal function was calculated using abbreviated MDRD^(8)^ formula and then staged according to the clinical practice guidelines published by the Working Group of the National Kidney Foundation^(11)^, as follows: stage 1 if GFR ≥90mL per minute; stage 2 if GFR from 60 to 89mL per minute; stage 3 if GFR from 30 to 59mL per minute; stage 4 if GFR from 15 to 29mL per minute; stage 5 if GFR <15mL per minute.

This research project was approved by the institutional Ethics Committee and registered under number 374,684. Due to the retrospective nature of the study, a consent form was not required for data collection.

## RESULTS

We identified 806 patients, but excluded 140 for incomplete information; thus 666 eligible patients were analyzed.

The median age was 74.2 years (range from 65 to 99 years) and 60% of the patients were male. Table 1 shows the most prevalent types of cancer in this patient population. Table 2 shows the distribution by clinical stage according to cancer type.

**Table 1:** Clinical characteristics of 666 elderly patients diagnosed with solid tumors in a cancer center

Clinical variables	Median (range)
Age (years)	74.2 (65-99)
Serum creatinine (mg/mL)	0.9 (0.4-4.5)
aMDRD (mL/min)	80 (19-166)
Types of cancer, n (%)	666 (100)
Colorectal	159 (24)
Prostate	136 (20)
Breast	105 (16)
Lung	104 (16)
Bladder	74 (11)
Pancreas	42 (6)
Gastric	28 (4)
Others*	18 (3)

*Central nervous system, thyroid, esophagus and gastrointestinal stromal tumor.

aMDRD: (Modification of Diet in Renal Disease)*.*

**Table 2:** Distribution by cancer stage according to primary site

Cancer type	Cancer stage				
	0	I	II	III	IV
**n (%)**	**n (%)**	**n (%)**	**n (%)**	**n (%)**
Colorectal, n=159	9 (6)	42 (26)	46 (29)	38 (24)	24 (15)
Prostate, n=136	0 (0)	4 (3)	106 (78)	16 (12)	10 (7)
Breast, n=105	11 (10)	41 (39)	29 (28)	17 (16)	7 (7)
Lung, n=104	0 (0)	28 (27)	10 (10)	21 (20)	45 (43)
Bladder, n=74	27 (36)	30 (40)	5 (7)	0 (0)	12 (17)
Pancreas, n=42	0 (0)	4 (10)	14 (33)	3 (7)	21 (50)
Gastric, n=28	0 (0)	5 (18)	4 (14)	6 (21)	13 (47)

**Figure 1:**
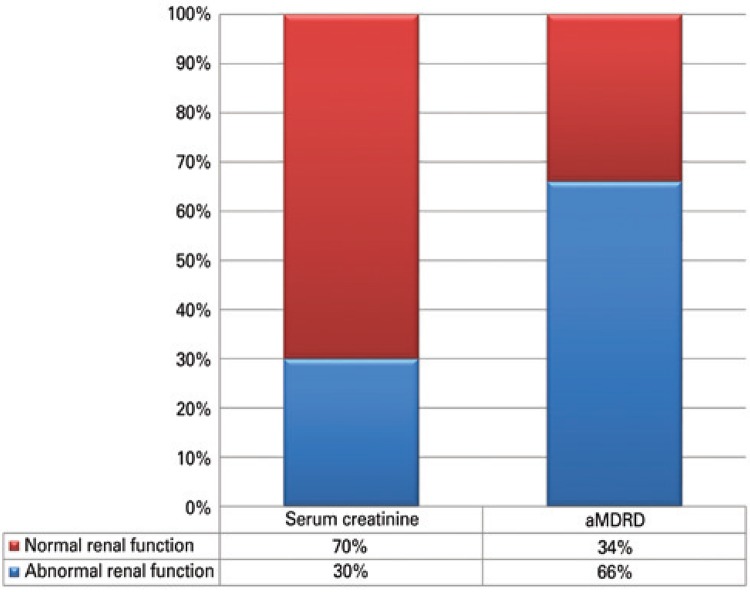
Comparison between serum creatinine levels and Modification of Diet in Renal Disease to estimate chronic kidney disease

**Figure 2:**
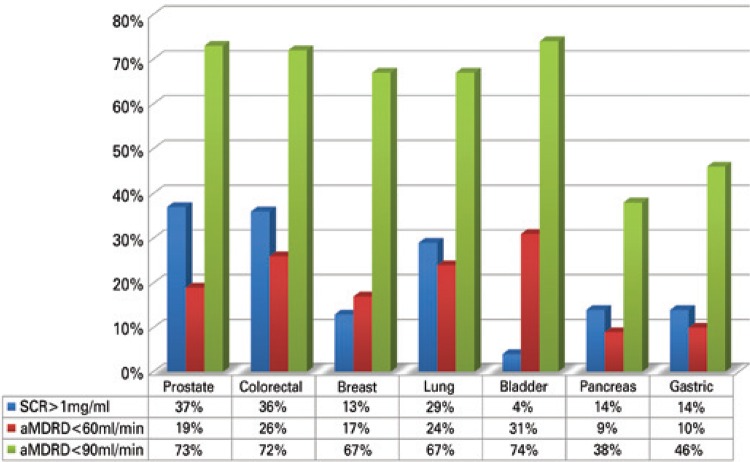
Chronic kidney disease for the main types of cancer

Out of studied patients, 30% (201/666) had an elevated SCR (>1.0mg/dL) at the time of diagnosis. However, when renal function was assessed using the abbreviated MDRD formula, 66% (439/666) had abnormal renal function (<90mL per minute). The cases with renal impairment were stratified as follows: 45% in stage 2, 18% in stage 3, 3% in stage 4 and 0.3% in stage 5. Figure 1 illustrates the comparison between the two methods to estimate RI in the elderly, showing the discrepancy between results. Figure 2 shows the percentage of patients with abnormal renal function by cancer type.

## DISCUSSION

Cancer therapy agents are frequently nephrotoxic, and some may not directly affect the kidneys but have different toxicities when renal excretion is not appropriate. These agents include chemotherapy drugs, molecular targeted therapy, pain medications and bisphosphonates.^(12)^ Thus, monitoring renal function in patients with cancer is critical for the safe administration of therapeutic agents, particularly in the group of elderly patients.

The prevalence of abnormal GFR as assessed by abbreviated MDRD among our cohort was high. Although we did not have information about preexisting comorbidities or other risk factors that could have increased the probability of renal impairment, the same pattern was observed by Janus et al.^(13)^ in a multicenter study in Belgium – the BIRMA study, in which 64% of patients had an abnormal renal function (GFR <90mL per minute) measured by the abbreviated MDRD formula upon initial diagnosis of cancer. Similar to our findings, the BIRMA study found only 12.5% of these patients with an elevated SCR. It is important to highlight that the patients included in our cohort were older than those studied by Janus et al.^(13)^ (mean age of 74.2 years *versus *61.3 years, respectively), which limits the value of comparisons.

We also found a high prevalence of decreased renal function in all tumors, regardless of the type, supporting the recommendation of universal evaluation of GFR. Comparable trends were also seen by Launay-Vacher et al.^(5)^, who described the prevalence of RI in 4684 cancer patients in France, and by Janus et al.^(13)^

Although we did not collect data on current treatment delivered to our patients, considering the types of cancers and the staging, we estimate that nearly 20% would have an indication for chemotherapy for metastatic disease and 22% for adjuvant treatment. Also, of the chemotherapic drugs that would be indicated according to standard treatment options, some are either nephrotoxic or would require dose adjustment due to low GFR, such as platinum compounds, pemetrexed and capecitabine (Chart 1). Probably, another small percentage of patients would require bisphosphonates, which also need dose adjustment. In the IRMA (RI and anticancer medications) study,^(5)^ for patients who had at least stage 2 RI, the frequency of nephrotoxic drug prescriptions was still high. In patients who had a creatinine clearance ranging from 60 to 90mL per minute, from 30 to 59mL per minute and from 15 to 29mL per minute, 53.6%, 60.2%, and 67% of anticancer drug prescriptions, respectively, were potentially nephrotoxic compounds. In clinical practice, it may not always be possible to avoid potentially nephrotoxic drugs, particularly when patients are being treated with curative intent; however, it is vital to be aware and to monitor.

**Chart 1:** Agents that must be used with caution based on renal function

Type of therapy	Agents
Chemotherapy^(14)^	Cisplatin, carboplatin, oxaliplatin, capecitabine, mitomycin, bleomycin, cabazitaxel, pemetrexed, cyclophosphamide, methotrexate, eribulin, etoposide, vinflunine, lomustine, dacarbazine, and carmustine
Molecular target therapies^(14,15)^	Crizotinib, imatinib, sorafenib, sunitinib, and vandetanib
IV bisphosphonates^(14,16)^	Pamidronate, and zoledronic acid

Some studies have demonstrated that pre-existing abnormal renal function is a risk factor for drug-induced nephrotoxicity,^(17)^ and this risk is already noticeable in patients with stage 2 RI (GFR: 60 to 89mL per minute). The risk of nephrotoxicity is even higher in patients with lower baseline GFR. The International Society of Geriatric Oncology (SIOG)^(16,18,19)^ has several recommendations regarding renal monitoring of elderly patients with cancer. Assessing comorbidities in geriatric patients is mandatory since treatment may have a significant impact on renal health, among other complications. It is strongly recommended to evaluate hydration status and renal function in all elderly cancer patients before administering drug therapy. In some cases, such as extremes of age, severe malnutrition or obesity and very high or low creatinine values, it is necessary to measure a 24-hour creatinine clearance to accurately estimate renal function.^(12,18)^

## CONCLUSION

To the best of our knowledge, this is the first study to estimate the frequency of renal insufficiency in elderly cancer patients in Brazil. Our results demonstrate that the prevalence of an abnormal renal function among elderly patients with cancer is high, and is most likely underestimated in clinical practice because of the use of serum creatinine levels as the standard evaluation. Thus, it is imperative that oncologists measure their patients’ renal function using alternative methods, such as abbreviated MDRD or creatinine clearance, before planning any therapeutic intervention.
