# Supplementary material for: Prevalence of renal insufficiency in elderly cancer patients in a tertiary cancer center
Source: Einstein (Sao Paulo). 2014 Jul-Sep;12(3):300–3. doi: 10.1590/S1679-45082014AO3003 (PMC4872939; doi:10.1590/S1679-45082014AO3003)
Supplement: Translation

# Translation: Prevalência de insuficiência renal em pacientes idosos com câncer em um centro de tratamento oncológico

**Authors:** Lucíola de Barros Pontes, Yuri Philippe Pimentel Vieira Antunes, Diogo Diniz Gomes Bugano, Theodora Karnakis, Auro del Giglio, Rafael Aliosha Kaliks

**Affiliations:** 1 Hospital Israelita Albert Einstein, São Paulo, SP, Brasil.; 2 MD Anderson Cancer Center, Houston, Texas, EUA.; 3 Faculdade de Medicina do ABC, Santo André, SP, Brasil.

**Keywords:** Insuficiência renal, Idoso, Neoplasias, Prevalência, Taxa de filtração glomerular

## Abstract

**Objetivo:**

Estimar a prevalência de taxa de filtração glomerular alterada em pacientes idosos diagnosticados com tumores sólidos.

**Métodos:**

Estudo retrospectivo de pacientes com mais de 65 anos de idade, diagnosticados com tumores sólidos entre janeiro de 2007 e dezembro de 2011 em um centro de tratamento oncológico. Foram coletados dados sobre sexo, idade, creatinina sérica à época do diagnóstico e tipo de tumor. A função renal foi calculada utilizando a versão simplificada da equação MDRD (*Modification of Diet in Renal Disease*) e depois estratificada de acordo com as diretrizes de prática clínica do *Working Group of the National Kidney Foundation*.

**Resultados:**

Foram incluídos 666 pacientes, sendo 60% do sexo masculino. A idade mediana foi 74,2 anos (variação de 65 a 99 anos), e os diagnósticos mais prevalentes na população do estudo foram câncer colorretal (24%), de próstata (20%), mama (16%) e pulmão (16%). A prevalência de creatinina sérica elevada (>1,0mg/dL) foi 30%. No entanto, quando os pacientes foram avaliados utilizando a forma abreviada da equação MDRD, 66% tinham uma função renal anormal assim estratificada: 45% em estádio 2, 18% em estádio 3, 3% em estádio 4 e 0,3% em estádio 5.

**Conclusão:**

Até onde sabemos, este foi o primeiro estudo a estimar a frequência de insuficiência renal em pacientes idosos com câncer no Brasil. A prevalência de função renal alterada na coorte estudada foi alta. Como suspeitávamos, o nível absoluto de creatinina subestima a alteração na função renal e não deve ser usado como preditor de metabolismo, excreção e consequente toxicidade dos agentes quimioterápicos.

## INTRODUÇÃO

A maior expectativa de vida levou a um aumento significativo na população de idosos. De acordo com o Instituto Brasileiro de Geografia e Estatística (IBGE),^(1)^ o Brasil tem 13 milhões de indivíduos com mais de 65 anos, e a expectativa é de que esse grupo alcance 39 milhões em 2040. Como mais de 60% de todos os tipos de câncer e 70% de todos os óbitos relacionados a câncer ocorrem em pacientes com mais de 65 anos,^(2)^ a população idosa representa um importante desafio em relação ao tratamento oncológico.

Ao escolher o tratamento apropriado para pacientes idosos, deve-se dar atenção especial a algumas alterações fisiológicas, como função renal reduzida, que predispõe os idosos a maior toxicidade da droga.^(3)^ A frequência da insuficiência renal (IR) em pacientes com câncer não está definida, e estudos mostram prevalência de 33 até 60%.^(4,5)^

Sabe-se que, em torno de 70 anos de idade, a função renal pode estar diminuída em 40%^(6)^ em relação aos valores iniciais. Desse modo, antes de se iniciar um tratamento medicamentoso potencialmente tóxico, como a quimioterapia, a função renal deve ser avaliada de forma rotineira. Os níveis de creatinina sérica (Cr) são usados na prática clínica em geral, mas não fornecem uma indicação precisa da função renal, especialmente em idosos, em que a IR é subestimada.^(7)^ Ferramentas mais acuradas e modernas, como a *Modification of Diet in Renal Disease* (MDRD),^(8)^ podem estimar melhor a função renal em pacientes idosos, como mostrado em alguns estudos, os quais propiciaram uma predição confiável da taxa de filtração glomerular (TFG) nessa população.^(9,10)^

Considerando-se que vários agentes quimioterápicos são eliminados pelos rins e podem interferir na função renal, e que a variabilidade na disfunção renal descrita é significante, procuramos estudar a prevalência de TFG anormal em pacientes idosos com diagnóstico de tumor sólido.

## OBJETIVO

Estimar a prevalência da taxa filtração glomerular anormal em pacientes idosos com tumores sólidos em um centro oncológico.

## MÉTODOS

Estudo retrospectivo que incluiu pacientes com idade de 65 anos ou mais diagnosticados com tumores sólidos entre janeiro de 2007 e dezembro de 2011 no Hospital Israelita Albert Einstein (HIAE), em São Paulo (SP), Brasil.

Coletamos as seguintes informações no banco de dados eletrônico da instituição: sexo, idade, peso, tipo de tumor e Cr no momento do diagnóstico. O limite superior de normalidade para Cr no laboratório central do HIAE é 1mg/dL. A função renal foi calculada pela forma abreviada da equação MDRD^(8)^ e, depois, classificada conforme as diretrizes de prática clínica, publicadas pelo *Working Group of the National Kidney Foundation*,^(11)^ quais sejam: estádio 1 se TFG ≥90mL por minuto; estádio 2 se TFG de 60 a 89mL por minuto; estádio 3 se TFG de 30 a 59mL por minuto; estádio 4 se TFG de 15 a 29mL por minuto; e estádio 5 se TFG <15mL por minuto.

Este projeto de pesquisa foi aprovado pelo Comitê de Ética da instituição e registrado sob número 374.684. Por ser um estudo retrospectivo, não foi necessário o termo de consentimento livre e informado para coleta de dados.

## RESULTADOS

Foram identificados 806 pacientes, mas 140 deles foram excluídos por informações incompletas. Assim, 666 pacientes foram analisados.

A mediana de idade foi 74,2 anos (variação de 65 a 99 anos), e 60% dos pacientes eram do sexo masculino. A tabela 1 mostra os tipos de câncer mais prevalentes nessa população de pacientes. A tabela 2 apresenta a distribuição por estádio clínico por tipo de câncer.

**Tabela 1:** Características clínicas de 666 pacientes idosos diagnosticados com tumores sólidos, em um centro de tratamento oncológico, em São Paulo, Brasil

Variáveis clínicas	Mediana (variação)
Idade (anos)	74,2 (65-99)
Creatinina sérica (mg/mL)	0,9 (0,4-4,5)
aMDRD (mL/min)	80 (19-166)
Tipos de câncer, n (%)	666 (100)
Colorretal	159 (24)
Próstata	136 (20)
Mama	105 (16)
Pulmão	104 (16)
Bexiga	74 (11)
Pâncreas	42 (6)
Gástrico	28 (4)
Outros*	18 (3)

*Sistema nervoso central, tireoide, esôfago e tumor gastrintestinal.

aMDRD: (Modification of Diet in Renal Disease).

**Tabela 2:** Distribuição por estádio de câncer conforme sítio primário

Tipo de câncer					
	0	I	II	III	IV
	n (%)	n (%)	n (%)	n (%)	n (%)
Colorretal, n=159	9 (6)	42 (26)	46 (29)	38 (24)	24 (15)
Próstata, n=136	0 (0)	4 (3)	106 (78)	16 (12)	10 (7)
Mama, n=105	11 (10)	41 (39)	29 (28)	17 (16)	7 (7)
Pulmão, n=104	0 (0)	28 (27)	10 (10)	21 (20)	45 (43)
Bexiga, n=74	27 (36)	30 (40)	5 (7)	0 (0)	12 (17)
Pâncreas, n=42	0 (0)	4 (10)	14 (33)	3 (7)	21 (50)
Gástrico, n=28	0 (0)	5 (18)	4 (14)	6 (21)	13 (47)

Dentre os pacientes, 30% (201/666) tinham Cr elevada (>1,0mg/dL) à época do diagnóstico. No entanto, quando a função renal foi avaliada pela forma abreviada da equação MDRD, 66% (439/666) tiveram função renal anormal (<90mL por minuto). Os casos com comprometimento renal foram assim classificados: 45% em estádio 2, 18% em estádio 3, 3% em estádio 4 e 0,3% em estádio 5. A figura 1 ilustra a comparação entre os dois métodos para estimar a IR em idosos e mostra a discrepância entre os resultados. A figura 2 mostra a porcentagem de pacientes com função renal anormal por tipo de câncer.

**Figura 1:** Comparação entre os níveis de creatinina sérica e a fórmula *Modification of Diet in Renal Disease*

**Figura 2:** Doença renal crônica e os principais tipos de câncer

## DISCUSSÃO

Os agentes para tratamento de câncer são, em geral, nefrotóxicos, e alguns podem não afetar diretamente os rins, mas apresentam diferentes toxicidades quando a excreção renal não é apropriada. Esses agentes incluem quimioterápicos, terapia de alvo molecular, analgésicos e bifosfonatos.^(12)^ Assim, o monitoramento da função renal em pacientes com câncer é essencial para a administração segura de agentes terapêuticos, especialmente no grupo de pacientes idosos.

A prevalência de TFG anormal, avaliada pela forma abreviada da MDRD, foi alta em nossa coorte. Apesar de não termos dados sobre comorbidades prévias ou outros fatores de risco que pudessem ter aumentado a probabilidade de dano renal, o mesmo padrão foi observado por Janus et al.^(13)^ em estudo multicêntrico na Bélgica – o estudo BIRMA, em que 64% dos pacientes tinham uma função renal anormal (TFG<90mL por minuto) medida pela forma abreviada da MDRD, à época do primeiro diagnóstico de câncer. De modo semelhante aos nossos achados, o estudo BIRMA encontrou apenas 12,5% desses pacientes com Cr elevada. Vale ressaltar que os pacientes incluídos nesta coorte eram mais velhos do que aqueles avaliados por Janus et al.^(13)^ (média de idade de 74,2 *versus* 61,3 anos, respectivamente), o que limita o valor das comparações.

Observamos também alta prevalência de função renal diminuída em todos os tumores, independentemente do tipo, o que corrobora a recomendação de avaliação universal da TFG. Tendências comparáveis foram apresentadas por Launay-Vacher et al.^(5)^, que descreveram a prevalência de IR em 4.684 pacientes com câncer, na França, e por Janus et al.^(13)^

Não foram coletados dados sobre o tratamento atual dado a nossos pacientes, considerando os tipos de câncer e o estadiamento, mas estimamos que aproximadamente 20% teriam uma indicação para quimioterapia para doença metastática e 22% para terapia adjuvante. Além disso, entre os quimioterápicos que poderiam ser indicados conforme as opções de tratamento padronizadas, alguns seriam nefrotóxicos ou necessitariam de ajuste de dose, devido à baixa TFG, como os compostos de platina, pemetrexede e capecitabina (Quadro 1). Provavelmente outra pequena porcentagem de pacientes necessitaria de bifosfonatos, que também requerem ajuste de dose. No estudo IRMA, sobre IR e medicações anticâncer,^(5)^ a frequência de prescrições de drogas nefrotóxicas ainda foi alta para pacientes que tinham IR grau 2, pelo menos. Para aqueles com *clearance* de Cr de 60 a 90mL por minuto, de 30 a 59mL por minuto e de 15 a 29mL por minuto, 53,6%, 60,2% e 67% de prescrições de medicamentos anticâncer, respectivamente, eram compostos potencialmente nefrotóxicos. Na prática clínica, nem sempre é possível evitar as drogas potencialmente nefrotóxicas, em especial quando os pacientes são tratados com propósito de cura. No entanto, é vital estar ciente disso e monitorar os pacientes.

**Quadro 1:** Agentes que devem ser usados com cautela em relação à função renal

Tipo de tratamento	Agentes
Quimioterapia^(14)^	Cisplatina, carboplatina, oxaliplatina capecitabine, mitomicina, bleomicina cabazitaxel, pemetrexede, ciclofosfamida, metotrexato, eribulin, etoposida, vinflunina, lomustina, dacarbazina e carmustina
Terapia de alvo molecular^(14,15)^	Crizotinibe, imatinibe, sorafenibe, sunitinibe e vandetanibe
Bifosfonatos IV^(14,16)^	Pamidronato e ácido zoledrônico

Alguns estudos demonstraram que a função renal prévia anormal é um fator de risco para nefrotoxicidade induzida por drogas,^(17)^ e esse risco já é observado em pacientes com IR grau 2 (TFG de 60 a 89mL por minuto). O risco de nefrotoxicidade é ainda maior em pacientes com TFG inicial mais baixa. A Sociedade Internacional de Oncologia Geriátrica (SIOG)^(16,18,19)^ tem diversas recomendações sobre monitoramento renal de pacientes idosos com câncer. A avaliação de comorbidades em pacientes geriátricos é obrigatória, pois o tratamento pode ter um impacto significativo na saúde renal, entre outras complicações. É altamente recomendado avaliar o estado de hidratação e a função renal em todos pacientes idosos com câncer antes de administrar o tratamento medicamentoso. Em alguns casos, como pacientes muito jovens ou muito idosos, desnutrição grave ou obesidade, e valores muito altos ou baixos de creatinina, é necessário medir o *clearance* de creatinina em urina de 24 horas para estimar com precisão a função renal.^(12,18)^

## CONCLUSÃO

Até onde sabemos, este foi o primeiro estudo que estimou a frequência de insuficiência renal em pacientes idosos com câncer no Brasil. Nossos resultados demonstram alta prevalência de função renal anormal em idosos com câncer, que é provavelmente subestimada na prática clínica devido ao uso de dosagens de creatinina sérica na avaliação padrão. Portanto, é imperativo que os oncologistas habituem-se a medir a função renal de seus pacientes com métodos alternativos, como MDRD ou o próprio *clearance*, antes de planejar quaisquer intervenções terapêuticas.
